# Comment on “The effect of preoperative embolization rate on surgical outcomes for carotid paraganglioma resection”

**DOI:** 10.1590/1806-9282.20241387

**Published:** 2024-12-02

**Authors:** Abdullah Güner, Muhammet Hüseyin Erkan

**Affiliations:** 1 Konya City Hospital, Department of Cardiovascular Surgery – Konya, Turkey.

Dear Editor,

We would like to share some thoughts on the study entitled “The effect of preoperative embolization rate on surgical outcomes for carotid paraganglioma resection”^1^.

This study aims to evaluate the effect of preoperative embolization of paragangliomas on surgical outcomes. The use of the Shamblin classification using preoperative contrast-enhanced neck CT images provided a standardized assessment of the anatomical characteristics of the tumors. A detailed description of preoperative embolization, the determination of the devascularization rate with the last angiogram performed after embolization, and a detailed evaluation of the efficacy of embolization are important. The calculation of tumor volume using both preoperative CT images and volumes measured after surgical resection shows that both the size of the tumor and the change after surgery were objectively assessed, and these criteria show the strong design of the study. However, we believe that it may be erroneous to compare a preoperative and a postoperative measurement, especially when evaluating the decrease in hemoglobin. Especially considering that the decrease in hemoglobin may be due to parenteral volume replacement before and during surgery in these patients, a decrease in hemoglobin may not only indicate bleeding. In the Methods and Results section of the study, the preoperative hemoglobin level was compared with the postoperative level, and there was no detailed explanation of the timing of the preoperative hemoglobin level. Similar to this issue, previous studies have generally considered the time of surgery, the amount of bleeding during surgery, and the need for postoperative blood transfusion when evaluating the effectiveness of preoperative embolization^2,3^. In this study, we believe that it would be more accurate to make a comparison by adding the time of surgery and the amount of bleeding during surgery instead of hemoglobin when evaluating the effectiveness of preoperative embolization.
